# Quantitative T1‐mapping detects cloudy‐enhancing tumor compartments predicting outcome of patients with glioblastoma

**DOI:** 10.1002/cam4.966

**Published:** 2016-11-28

**Authors:** Andreas Müller, Alina Jurcoane, Sied Kebir, Philip Ditter, Felix Schrader, Ulrich Herrlinger, Theophilos Tzaridis, Burkhard Mädler, Hans H. Schild, Martin Glas, Elke Hattingen

**Affiliations:** ^1^ Neuroradiology, Department of RadiologyUniversity Hospital BonnSigmund Freud Str. 25Bonn53127Germany; ^2^ Division of Clinical NeurooncologyDepartment of NeurologyUniversity Hospital BonnSigmund Freud Str. 25Bonn53127Germany; ^3^ Philips GmbHUB HealthcareLübeckertordamm 5Hamburg20099Germany; ^4^ Division of Experimental and Translational NeurooncologyDepartment of NeurologyUniversity Hospital BonnSigmund Freud Str. 25Bonn53127Germany; ^5^ Clinical Cooperation Unit NeurooncologyMediClin Robert Janker Clinic & University Hospital BonnSigmund Freud Str. 25Bonn53127Germany

**Keywords:** Cloudy‐enhancing compartment, glioblastoma, progression‐free survival PFS, quantitative MRI, T1‐mapping

## Abstract

Contrast enhancement of glioblastomas (GBM) is caused by the decrease in relaxation time, T1. Here, we demonstrate that the quantitative measurement of T1 (qT1) discovers a subtle enhancement in GBM patients that is invisible in standard MRI. We assessed the volume change of this “cloudy” enhancement during radio‐chemotherapy and its impact on patients’ progression‐free survival (PFS). We enrolled 18 GBM patients in this observational, prospective cohort study and measured 3T‐MRI pre‐ and post contrast agent with standard T1‐weighted (T1w) and with sequences to quantify T1 before radiation, and at 6‐week intervals during radio‐chemotherapy. We measured contrast enhancement by subtracting pre from post contrast contrast images, yielding relative signal increase ∆T1w and relative T1 shortening ∆qT1. On ∆qT1, we identified a solid and a cloudy‐enhancing compartment and evaluated the impact of their therapy‐related volume change upon PFS. In ∆qT1 maps cloudy‐enhancing compartments were found in all but two patients at baseline and in all patients during therapy. The qT1 decrease in the cloudy‐enhancing compartment post contrast was 21.64% versus 1.96% in the contralateral control tissue (*P* < 0.001). It was located at the margin of solid enhancement which was also seen on T1w. In contrast, the cloudy‐enhancing compartment was visually undetectable on ∆T1w. A volume decrease of more than 21.4% of the cloudy‐enhancing compartment at first follow‐up predicted longer PFS (*P* = 0.038). Cloudy‐enhancing compartment outside the solid contrast‐enhancing area of GBM is a new observation which is only visually detectable with qT1‐mapping and may represent tumor infiltration. Its early volume decrease predicts a longer PFS in GBM patients during standard radio‐chemotherapy.

## Introduction

Glioblastomas (GBM) are highly vascular tumors that overexpress the vascular endothelial growth factor (VEGF)^1^ which promotes neoangiogenesis with pathological vessels and leaky blood–brain barrier (BBB). T1‐weighted (T1w) MRI post contrast agent is the clinical mainstay in routine clinical imaging to detect BBB breakdown 2. Gadolinium‐containing contrast agent shortens the relaxation time T1 3, 4, 5 when it accumulates in brain or tumor tissue outside the vessels. Therefore, impaired BBB is seen as hyperintense, enhanced regions on postcontrast T1w images. Subtraction of postcontrast T1w images from precontrast images 6, 7 increases visibility of enhancement which allows better assessment of its volume. This assessment may be essential for GBM surgery since complete resection of the enhancing part of GBM improves patient's prognosis 8. Ellingson et al. evaluated multicenter MRIs of patients with recurrent GBM under antiangiogenic therapy. They could show that the contrast‐enhancing tumor volume defined by subtraction of postcontrast T1w images predicts clinical outcome in patients with recurrent GBM under antiangiogenic therapy 9. However, due to inherent signal inhomogeneities in T1w images, they had to correct the T1w images for these effects before subtracting them.

T1‐relaxometry is a method which a priori considers inhomogeneities which influence the T1‐effects. It quantifies T1 relaxation time (qT1) in milliseconds for each brain voxel both before and after contrast agent. Therefore, it not only visualizes, but also quantifies the BBB disruption 10, 11. Quantification allows comparison across subjects, time points, and imaging centers 12, 13, an advantage against the weighted images 14. However, the clinical impact of this T1‐mapping to evaluate enhancing tumor volume is not evaluated yet.

In this study, we compared the detectability of BBB breakdown in weighted images and quantitative T1 mapping in patients with GBM. We describe a new contrast‐enhancing compartment only obvious in the quantitative imaging and analyze the prognostic implications of this new tumor compartment.

## Materials and Methods

### Patient population and study protocol

All patients participating in this observational, noninterventional cohort study signed an institutional review board‐approved informed consent prior to enrolment. The recruitment was determined for 9 months (from December 2014 to August 2015) to reach a patient number of approximately 20 patients. Eligibility criteria were treatment‐naïve patients with histopathologically confirmed diagnosis of primary GBM or gliosarcoma. To keep the loss of follow‐up of patients small, we only included patients with a Karnofsky Performance Score more than 70%. We also set value on a strong family support to ensure the regular participation of the patients.

MRIs were acquired after tumor surgery and before radiotherapy onset—baseline time point 0 (TP0), every 6 weeks (TP1, TP2, TP3 etc.) during radiotherapy or when therapy changed (corticosteroids, chemotherapy, or renewed surgery) with a median of six MRIs per patient (range: 5–10). Patients missing one TP were not excluded from the study. Data from patients who did not reach TP5 were excluded from analysis.

### MRI study protocol

We used a 3.0 Tesla whole body MR‐scanner (Achieva, Philips Healthcare, The Netherlands) with an 8‐channel phased array head coil. We acquired T1w sequences, sequences to map qT1, dynamic susceptibility‐weighted (DSC) perfusion, 2D Fluid‐attenuated inversion recovery (FLAIR), and T2‐weighted turbo spin echo sequences.

T1w sequence was a 2:07 min whole‐brain spin echo sequence with 728 msec repetition time, 13.4 msec echo time, 90° flip angle, 250 × 190 mm^2^ field of view, and 28 slices of 5 mm thickness and 1 mm interslice gap.

For qT1 mapping, we used a 8:20 min whole‐brain 3D Inversion recovery (IR) prepared ultrafast gradient echo (TFE) sequence and following parameters: isotropic resolution 1 × 1 × 1 mm^3^, shortest repetition time (4.81–5.07 msec), echo time 2.39–2.49 msec, flip angle 15°, five IR delays (inversion times 150, 350, 750, 1200, 2300 msec) with a shot interval of 3000 msec, field of view 240 × 220 mm^2^ and 120 slices.

Both T1w and qT1 sequences were conducted pre‐ and post intravenous administration of Gd‐DO3A‐butrol (Gadovist^®^, Bayer Vital GmbH) of 0.1 mmol/kg body weight. To exclude cloudy enhancement being an effect of “late enhancement”, in three exemplary cases, we acquired postcontrast T1w sequences before—as in all patients—as well as after the T1‐mapping.

### Postprocessing of quantitative imaging data

We generated maps of T1‐relaxation time from IR‐magnitude data, with a fixed likelihood estimate for the goodness of the inversion pulse (F‐factor), accounting for the incomplete longitudinal relaxation at the next excitation. To remove noise from the slight enhancement of the normal‐appearing tissue reported in both patients and controls 15, 16, we scaled the qT1 and T1w pre‐ and post contrast relative to the mean in a control region in the normal‐appearing white matter.

We generated subtraction maps based on the coregistered quantitative T1‐maps (∆qT1) or weighted T1 images (∆T1w) pre and post contrast as follows:

$$\begin{array}{cc} & \text{T1}\;\text{subtraction map}(\Delta \text{T1})=\\ & \;100*\frac{\left|\text{T1}(\text{precontrast})-\text{T1}(\text{postcontrast})\right|}{\text{T1}\;(\text{precontrast})} \end{array}$$

Fitting was done with an in‐house script in Matlab (release 2014a, MathWorks Inc), image arithmetic/statistics was done with fslmaths and fslstats (both part of FSL, Oxford UK, http://fsl.fmrib.ox.ac.uk) and linear coregistration was done with reg_aladin (Translational Imaging Grup, http://cmictig.cs.ucl.ac.uk/wiki/index.php/Reg_aladin).

### Volumetric assessment of enhancement

**Figure 1:**
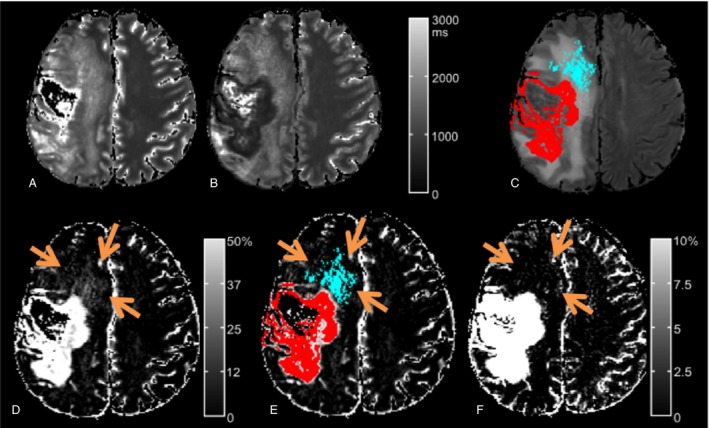
Quantitative T1 map of patient N 3 (male, age 67). T1 maps pre (A) versus post (B) contrast agent show the T1‐shortening due to the impaired BBB induced by the tumor. The subtraction map ∆qT1 (D) not only delineates the solid‐enhancing tumor compartment (red in C and D), but also areas with cloudy enhancement in the vicinity of the solid tumor (blue in C and D) that is inside, but not congruent with the FLAIR hyperintensity (C). This cloudy‐enhancing compartment is not visible on conventional T1‐weighted subtraction images (F) regardless of the window width chosen for display. Even more, at a low threshold window (note scale in F), noise becomes visible in the whole white matter and in the necrosis. The semiautomated volumetry defined cloudy‐enhancing compartment (blue) by a T1‐decrease of 10–50% and solid‐enhancing compartment (red) by T1‐decrease of more than 50% of T1‐decrease postcontrast. FLAIR, Fluid‐attenuated inversion recovery.

On ∆qT1 maps, we semiautomatically identified the contrast‐enhancing compartments with ITK‐SNAP 17 using the “Thresholding” mode, which applies a user‐guided 3D active contour algorithm to expand user‐defined seed regions, and we manually excluded partial volume effects from vessels, cortices, or ventricular borders. We defined:

1. Solid‐enhancing compartment as area with >50% T1 shortening
2. A cloudy‐enhancing compartment as area with 10–50% T1 shortening excluding the partial volume in the margins of the solid enhancement (Fig. 1).

Thus, at each TP, we measured the ∆qT1 and ∆T1w volumes (and volume overlap—Dice similarity index) of cloudy‐enhancing compartment and the complete solid‐enhancing volume including areas “not measurable” according to RANO criteria 18 (e.g., due to large necrosis).

The cloudy‐enhancing compartment occurred outside the obvious solid enhancement, within, but not completely congruent with theT2/FLAIR hyperintensity (Fig. 1).

### Cerebral blood volume of the cloudy‐enhancing compartment

We further assessed the regional cerebral blood volume (rCBV) of the cloudy‐enhancing compartment to get more biological information on this first described phenomenon.

The rCBV was calculated using a software tool (perfusion tool of Intellispace 9, Philips^®^) that additionally corrects confounding T1‐effects of contrast agent that potentially passes the pathologically permeable blood vessels. This program fits and adapts the signal‐time curve of each voxel to correct mathematically the leakage (extravasation correction) using a linear fitting algorithm 19. We extracted rCBV_mean_ from the area of the cloudy‐enhancing compartment and from the contralateral corresponding brain tissue, both regions excluding vessels and CSF for each patient, at the TP with the most pronounced cloudy compartment.

### Assessment of tumor progression

Progressive disease was not diagnosed within the first 3 months after the end of radiotherapy. As per RANO criteria 18, progression was defined by clinical worsening or by >25% increase in the sum of diameters of all measurable T1w contrast‐enhancing lesions or by new contrast‐enhancing lesion(s) outside the 80% isodose radiation field or by histopathological proof. MR images were independently analyzed by two experienced neuroradiologists (both board‐certified, each with 8 and 14 years of experience, respectively). Due to the 6‐week interval between the MR scans, there was no MRI performed at exactly 6‐months; therefore, we analyze here the progression‐free survival (PFS) at TP5 (about 7 months after baseline).

### Statistics

We compared regional changes upon contrast agent administration through therapy with two‐sided, unpaired Student's t tests (qT1 against T1w, solid compartment vs. cloudy compartment, rCBV_mean_ in the cloudy compartment against the contralateral rCBV_mean_). We evaluated the impact of an early (TP0 to TP1) decrease in the cloudy‐enhancing compartment on PFS with the Kaplan–Meier estimator with the log‐rank test and determined the optimal cutoff value with receiver operating characteristics (ROC) analysis, using PFS at TP5. We also tested if the decrease in the cloudy enhancement volume correlated with the tumor MGMT (O6‐Methyl‐Guanine‐Methyl‐DNA‐Transferase promoter methylation) promoter status with Fisher's exact test. We used R Statistics (v.3.2.5, 2016, http://www.R-project.org/) and SPSS and set significance at *P* < 0.05. Unless otherwise stated, values are given as mean ± standard deviation and 95% confidence interval (CI).

## Results

### Patients

**Figure 2:**
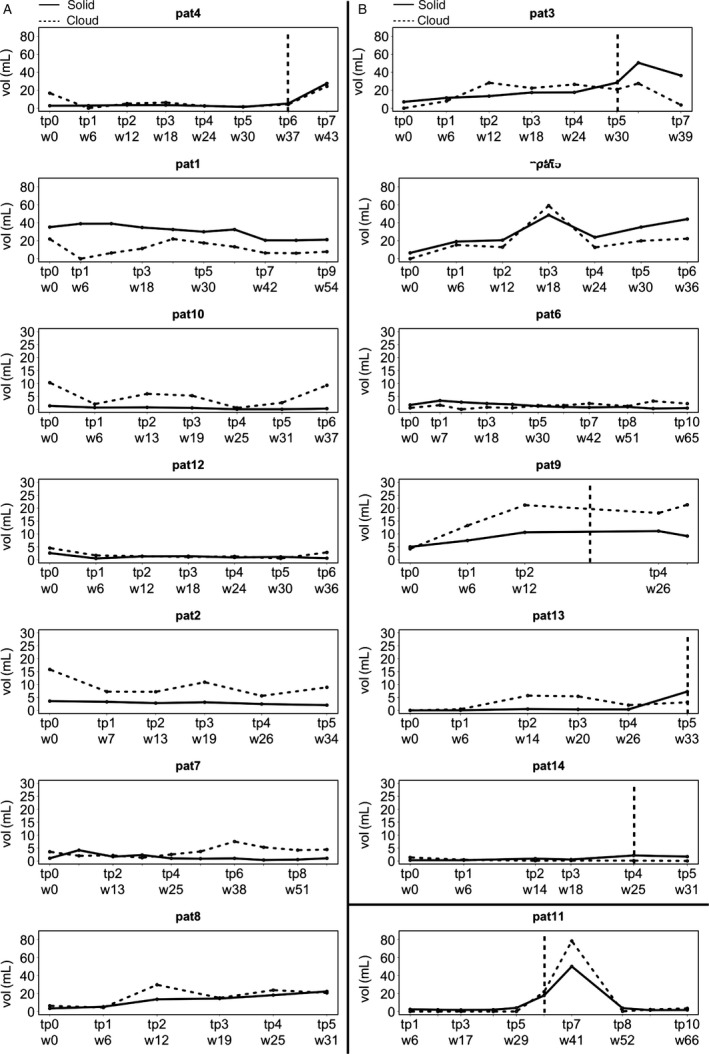
Volumes of cloudy‐enhancing and solid‐enhancing compartments during treatment. Patients with >21.4% decrease in the cloudy‐enhancing compartment at first follow‐up (A), and patients with <21.4% decrease in the cloudy‐enhancing compartment at first follow‐up (B). TP of tumor progression according to RANO is indicated with a dotted vertical line. Note, that patients of group A had a progression‐free survival (PFS) of at least 7 months (TP5). In contrast, most of the patients of group B had shorter PFS. The necrotic tumor of patient 5 was not progressive according to RANO because it was not measurable.

Eighteen patients (mean age 58 years, range: 39–76) were successively enrolled in this prospective noninterventional study (total number of time points = 107). Seven of the patients were female. Four patients received a stereotactic biopsy. All but one patient received concomitant radio‐chemotherapy (details are given in Table 1), followed by adjuvant temozolomide chemotherapy according to the Stupp protocol 20. Mean Karnofsky Performance Score was 90 ± 9.25% (standard deviation). Out of the 18 patients recruited, one patient died from pulmonary embolism after TP2, one patient with a huge bifrontal GBM died before TP2 and his MR scans showed severe motion artifacts, one patient did not fit in the scanner due to steroid‐induced weight gain after the first time point and one patient had severe motion artifacts at several time points. These four patients were excluded from the study and the final analysis included the remaining 14 patients who all reached at least TP5. Patients were imaged every 6 weeks or upon therapy changes (e.g., Pat. No. 9 in Fig. 2) and the total follow‐up time was on average 7.66 ± 0.35 months, 95% CI = [7.46–7.86 months] after baseline. Patient characteristics and treatment details are shown in Table 1. PFS time for each patient according the RANO criteria is included.

**Table 1:** Data from patients included in the analysis: Patients’ characteristics, treatment details, volume of cloudy‐enhancing compartment at time points (TP) 0 (baseline before starting therapy) and at TP1 (6 weeks later, toward the end of radiation), and volumes of the solid‐enhancing compartment (solid) at TP5 (about 7 months after baseline)

Pat. No.	Age (year), gender	First‐line therapy	MGMT promoter status	Radiation dose (Gy)	Dexamethasone treatment	Follow‐up time (week, TP)	PFS (months)	TP with progression	Cloud TP0 (mL)	Cloud TP1 (mL)	Cloud % change TP0‐TP1	Solid TP0 (mL)	Solid TP5 (mL)	Progression at TP5
1	53, f	B, RT + TMZ, TMZ (6 cy) + TTF	N	22	3.5–8 mg as of surgery	54, TP9	13.8	n.a.	21.95	2.30	−89.5	35.20	30.01	no
2	76, m	cR, RT + TMZ, TMZ (5 cy)	P	60	–	34, TP5	10.3	n.a.	15.84	7.26	−54.2	3.60	2.05	no
3	67, m	pR, RT + TMZ, TMZ (5 cy), +CCNU after TP6	N	60	8 mg/day at progression	39, TP7	7.4	TP5	0.00	7.85	infinite	7.03	28.45	yes
4	62, f	pR, RT + TMZ, TMZ (5 cy)	N	60	–	43, TP7	8.8	TP6	16.75	0.00	−100	2.76	1.44	no
5	50, m	B, RT + TMZ, TMZ (5 cy)	P	60	10–12 mg as of surgery	36, TP6	8.7	n.a.	0.00	15.34	infinite	6.51	35.19	no^a^
6	40, f	cR, RT+TMZ, CCNU + TMZ (6 cy)	P	60	4 mg peri‐operatively	65, TP10	16.4	n.a.	0.54	1.61	196.5	1.67	1.22	no
7	46, m	cR, RT+TMZ, CCNU + TMZ (6 cy)	P	60	–	57, TP9	14	n.a.	3.56	1.99	−44.0	1.04	0.88	no
8	74, m	pR, RT + TMZ, TMZ (4 cy)	P	40.05	8 mg peri‐operatively	31, TP5	7.6	n.a.	6.38	4.43	−30.5	3.44	22.35	no^a^
9^a^	59, m	B, RT + TMZ, TMZ (3 cy)	N	60	40 mg since TP5	30, TP5	5.4	TP3	4.28	13.27	209.8	5.03	9.20	yes
10	52, f	pR, RT + TMZ + CCNU, TMZ+CCNU (5 cy)	P	60	8 mg/day while under RT	37, TP6	9.3	n.a.	10.30	2.02	−80.4	1.33	0.02	no
11^a^	61, m	cR, RT + TMZ, TMZ (2 cy)	N	60	8 mg/day at progression	60, TP10	10.5	TP6	NA	0.00	NA	NA	NA	no
12	55, m	B, RT + TMZ + CCNU, TMZ+CCNU (6 cy)	P	60	–	36, TP6	9.3	n.a.	4.57	1.72	−62.5	2.63	1.17	no
13	72, f	cR, RT mono, TMZ (4 cy)	P	40.05	12 mg/day at progression	33, TP5	8.6	TP5	0.01	0.47	9200	0.00	7.29	yes
14	69, f	pR, RT + TMZ, TMZ (4 cy)	N	60	8 mg/day prior to surgery	31, TP5	6.4	TP4	2.84	2.49	−12.2	0.30	1.71	yes

Shadowed rows represent patients with >21% decrease in the cloudy‐enhancing compartment at first follow‐up. f, female; m, male; B, stereotactic biopsy; cR, complete resection; pR, partial resection; RT, radiation therapy; cy, cycles; TMZ, temozolomide; CCNU, Chlorethyl‐Cyclohexyl‐Nitroso‐Urea; *MGMT promoter status, O6‐Methyl‐Guanine‐Methyl‐DNA‐Transferase* promoter methylation; P, positive; N, negative; TTF, tumor treating field; steroid, dexamethasone; TP, time point; n.a., not applicable. Cloud = cloudy‐enhancing compartment defined by 10–50% T1‐decrease and solid= solid‐enhancing compartment defined by >50% of T1‐decrease after intravenous injection of Gadolinium‐containing contrast agent.

Not measurable large necrotic lesions.

New spinal lesion 1 month after TP2; could not attend TP3, new brainstem lesion at TP4.

a Patient 11 had no measurement at TP0 and underwent second resection after TP7.

From the 14 patients reaching TP5, six had progressive disease during follow‐up according to RANO. Two of the patients without progression had tumors with growing necrotic volumes and only marginal, nonmeasurable enhancement areas. However, in these patients the semiautomated volumetry of the solid‐enhancing compartment on ∆qT1 maps revealed also an increase over time (Fig. 2, patients No 5 and No 8).

### Contrast enhancement

The contrast enhancement visible on ∆T1w was equally visible on ∆qT1 maps as a solid enhancement compartment and had 90% volume overlap (similarity coefficient) between modalities.

Visually, the cloudy‐enhancing compartment could not be detected on ∆T1w maps due to the marginal difference from normal tissue (Fig. 1). This was also true for the three exemplary cases, for which the postcontrast T1w images were acquired after the T1‐mapping sequences, which excluded that the cloudy‐enhancing compartment is only an effect of “late enhancement”.

**Figure 3:**
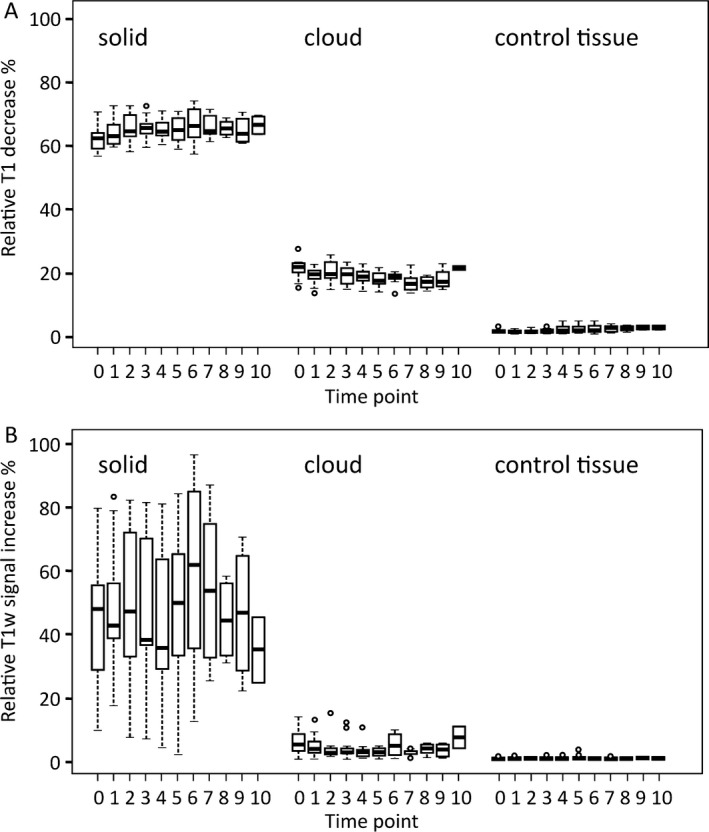
Relative decrease in relaxation time T1 (a) and relative increase in T1 signal intensity (b) upon intravenous injection of contrast agent, measured on the respective subtraction maps ∆qT1 and ∆T1w (boxes: first and third quartiles; thick lines: median; whiskers: most extreme data values excluding outliers; circles: outliers).

Quantitative MRI at TP0 could not be acquired in one patient. At TP0 11/13 patients and at TP1 12/14 patients showed cloudy‐enhancing compartments outside the solid‐enhancing tumor compartment. Three exemplary cases are shown in the Figure S1. The shortening of T1 on qT1 maps at TP0 was ∆qT1 = 21.64 ± 3.44%, 95% CI = [19.32, 23.95%] in the cloudy‐enhancing compartment versus ∆qT1 = 1.96 ± 0.46%, 95% CI = [1.68, 2.24%] in contralateral control regions (*P* < 0.001). In the T1w subtraction maps the signal intensity of the identical regions was ∆T1w = 6.54 ± 4.23%, 95% CI = [3.7–9.38%] in the cloudy‐enhancing compartment versus ∆T1w = 0.83 ± 0.18%, 95% CI = [0.73–0.94%] in contralateral control regions (*P* < 0.001). In the follow‐up, each patient had a cloudy‐enhancing compartment at least for one time point (Fig. 2). The median values of ∆qT1 in the cloudy‐enhancing compartment were around 20% for all time points, and values of ∆T1w fluctuated around 5% (Fig. 3). The values of signal intensities in the ∆T1w images had also larger standard deviations (range 3–6% for ∆qT1 vs. 14–29% for ∆T1w, *P* < 0.05 at any time point).

For each time point the mean relative T1 change in the solid‐enhancing compartment was significantly larger in the ∆qT1 maps than in the ∆T1w images (range 62–67% vs. 35–58%, *P* < 0.05 at any time point) (Fig. 3). At all time points, the values in the control VOI varied between 1.8% and 3.2% in the ∆qT1 maps and between 0.8% and 1.1% in the ∆T1w images (*P* < 0.05 at any time point).

### Prognostic value of the cloudy‐enhancing compartment

**Figure 4:**
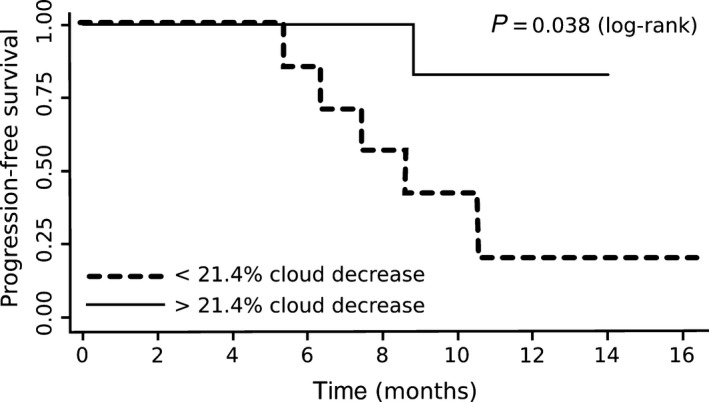
Kaplan–Meier estimator for PFS for patients with a > 21.4% decrease in cloudy‐enhancing compartment at first follow‐up (TP1) compared to baseline (solid line) and patients with a < 21.4% decrease in cloudy‐enhancing compartment at first follow‐up compared to baseline (dotted line). PFS, progression‐free survival.

ROC analysis revealed that a volume decrease in the cloudy‐enhancing compartment of more than 21.4% at TP1 predicts a longer PFS at TP5 with a sensitivity of 100% and a specificity of 77.8% (*P* = 0.034, AUC 92.6 ± 8.4%). Seven of the 13 patients had an early and marked volume decrease in the cloudy‐enhancing compartment in the first follow‐up (TP1), which means 6 weeks after starting radiotherapy with concomitant temozolomide. Using a cutoff value of 21.4%, Kaplan–Meier analysis revealed that patients with a volume decrease in the cloudy‐enhancing compartment of more than 21.4% have a longer PFS than those without such decrease (Kaplan–Meier, log–rank test, *P* = 0.038) (Fig. 4).

Six of the seven patients with a volume decrease in the cloudy‐enhancing compartment more than 21.4% had a lower volume of the solid‐enhancing compartment at TP5 compared to baseline (Table 1, Fig. 2). The decrease in the cloudy enhancement volume did not correlate with the MGMT promoter status of the respective tumors (*P* = 0.592).

ROC analysis of the volume change in the solid‐enhancing compartment at TP1 did not reveal a cutoff value that predicted better PFS for the patients at TP5 (*P* = 0.5185, AUC 51,9 ± 16.7%).

### Cerebral blood volume of the cloudy‐enhancing compartment

In all but one subject, the rCBV_mean_ was higher in the cloudy‐enhancing compartment compared to the rCBV_mean_ of the normal‐appearing contralateral white matter. The ratio between the two regions was significantly different from 1 (*P* < 0.01, 95% CI = 1.16–1.43).

The one patient with a lower rCBV_mean_ in the cloudy‐enhancing compartment had a subtotal tumor resection and was stable over time with a < 5 mL residuum of solid enhancement. The volume of the cloudy‐enhancing compartment was also small (15 mL) at TP0 and decreased markedly from TP0 to TP1 (5 mL).

## Discussion

Cloudy enhancement outside the solid contrast‐enhancing area of GBM is a new observation that is only visually detectable with qT1‐mapping. Areas with subtle “cloudy” contrast enhancement were detected in all GBM patients under treatment. This phenomenon has clinical implications, since an early reduction in this cloudy‐enhancing compartment under therapy predicts a favorable therapy response.

An early decrease of more than 21.4% of this cloudy‐enhancing compartment was prognostic for a favorable therapy response, which takes effect just after the first weeks after starting the treatment, even before pseudoprogression might occur. On the other hand, persistence of this cloudy‐enhancing compartment under therapy seems to be related to the presence and persistence of vital tumor. In contrast, changes of the solid‐enhancing tumor after beginning of therapy were not related to patients’ outcome.

Subtracting contrast‐enhanced from noncontrast‐enhanced T1w images has been shown to be predictive for patient survival in recurrent GBM under antiangiogenic therapy 9. Our work shows that quantitative subtraction maps discover a cloudy‐enhancing compartment in GBM which appears beyond the enhancing tumor and which is not visually detectable with weighted subtraction images.

The phenomenon of cloudy‐enhancing tumor areas is new. It is not a result from “late enhancement” since the conventional T1 w images—recorded after the T1‐mapping sequences—failed to show the cloudy enhancement. Unlike quantitative T1‐mapping, which measures the T1‐shortening without any confounding effects, conventional T1w images are influenced by B1 field inhomogeneities and contain mixed contrasts influenced by T1 and T2* relaxation times and the proton density.

The cloudy‐enhancing compartment seems to be an early discriminator between therapy response and therapy failure. A marked volume decrease in this cloudy‐enhancing compartment of at least 21.4% just 6 weeks after starting therapy is prognostic for longer PFS. In these cases, the volume decrease in cloudy‐enhancing compartment may be interpreted as an early response to radio‐chemotherapy. The other way around, patients with increasing or persistent cloudy‐enhancing compartments have a shorter PFS and an increase in solid‐enhancing volume about 7 months later (negative predictive value 83%). These results also strongly suggest that the cloudy‐enhancing compartment is directly tumor‐associated. The high sensitivity to treatment might be explained by the lesser tumor burden in these tumor infiltration zones, which might be easier to reduce with radiation and/or chemotherapy. Interestingly, the decrease in the cloudy‐enhancing compartment did not correlate with the MGMT status of the respective tumors.

The contrast‐enhancing part of the glioblastomas on conventional MRI represents the tip of the iceberg since it does not delineate the real tumor borders. The diffuse infiltration of the glioma cells beyond the MR visible tumor borders is one of the most important characteristics of the glioblastoma, making it difficult to plan the borders for tumor resection or radiation 21. To improve the delineation of the real tumor borders, the peritumoral region of glioblastomas outside the MR‐enhancing tumor margins has been investigated with other functional MR methods such as MR spectroscopy, diffusion tensor imaging (DTI), and with MR spectroscopy 21. In MR spectroscopy, the NAA decreases 22 or the choline‐to‐N‐acetyl‐aspartate index 23 indicated glioma cell infiltration in the peritumoral region, whereas in DTI, the decrease in FA values was a more unreliable sign of infiltration 24. The best investigated parameter of tumor infiltration is the rCBV measured with dynamic susceptibility‐weighted perfusion 25, 26, 27, 28. An increase of rCBV in gliomas correlated well with their pathological neovascularization 2, so that peritumoral rCBV increase was also thought to indicate vascular changes induced by tumor infiltration 27, 28. In line with other authors, we found an increase of rCBV in the peritumoral cloudy‐enhancing compartment for all but one patient. Considering that normal edema rather shows a decrease in rCBV in the absence of glioma infiltration 25, 29, our finding strongly supports the hypothesis that the cloudy‐enhancing compartment reflects the adjacent infiltration zone with cooption of normal vasculature and/or neoangiogenesis.

Tumor VEGF may diffuse in the adjacent normal brain and it is even present in plasma; thus, it can distribute ubiquitously throughout the brain, but for developing its effects, docking on special VEGF receptors on tumor cells is needed 30. Therefore, the presence of tumor cells seems to be mandatory to explain persistence or increase in the cloudy‐enhancing compartment. Its focal nature and its common simultaneous volume increase with the solid‐enhancing tumor also speak for the presence of tumor‐induced angiogenesis. This phenomenon is also called the angiogenetic switch 31, which means that under certain pathological conditions, for example, during tumor progression, the action of positive regulators predominates, and angiogenesis is active.

The cloudy enhancement extended as far as several centimeters from the solid tumor component. One could argue that the cloudy enhancement is a phenomenon of diffusing gadolinium‐chelate‐molecules from the solid‐enhancing tumor into the surrounding tissue and that this “dilution” causes the cloudy enhancement. The large diameter of the chelate complexes in the dimensions of nm makes this hypothesis unlikely, as the time between injection of contrast and image acquisition is too short to allow a diffusion of these molecules over such distances 32. In addition, in our study, the cloudy enhancement was within, but not completely congruent with the T2/FLAIR hyperintensity, which also argues against cloudy‐enhancing compartment as remote phenomenon of the solid tumor mass (see also Fig. 1).

However, some diffusion of gadolinium‐chelate‐molecules from the tumor bed might play a role in cloudy‐enhancing compartments, maybe explaining lower rCBV value in one of our patients.

Therefore, subtraction maps of qT1 measurements seem to expose a new compartment with subtle tumor‐associated damage of the BBB. The advantage of quantitative methods over other MR methods is that they not only directly visualize the peritumoral tumor infiltration but, being high‐resolution 3D images, also allow delineating and measuring the volume of this infiltration. This high‐resolution 3D visualization of tumor extension may help to plan the radiation field or to monitor the tumor infiltration.

### Limitations

The phenomenon of the cloudy‐enhancing compartment requires further investigations, especially histopathological assessment. It was not ethical in this study to get a biopsy of the cloudy‐enhancing compartment without clinical implications. Due to the small patient number our statistical analysis only gives unadjusted estimates. The low patient number was due to the restricted inclusion criteria to avoid loss of follow‐up. The 6‐week period of MR examinations required high compliance of patients and their families. Therefore we did not reach the intended patient number of about 20 during the recruitment period of 9 months.

In conclusion we describe a new tumor compartment only detected by T1 mapping and not seen in standard MR imaging. This new tumor compartment is characterized by a cloudy enhancement pattern and may represent diffuse infiltrating tumor. Our study shows that monitoring of this compartment detects early and sensitive therapy responses, since a marked volume decrease of this compartment at first follow‐up is prognostic for a longer PFS in GBM patients.

## Conflict of Interest

None declared.

## Supporting information

Figure S1. A. Representative ∆qT1 maps at baseline (TP0) and seven follow‐ups (TP 1‐7) of patient No 3 (male, age 67). Patient had a residual solid‐enhancing tumor volume in the right central region, which increased continuously during therapy. The cloudy‐enhancing compartment firstly occurred at TP1 and increased again at TP2. During follow‐up, the cloudy‐enhancing compartment persisted and the solid‐enhancing compartment increased steadily until therapy was intensified with CCNU at week 33 (marked with * after TP6). Its persistence during tumor progression, and its simultaneous decrease with the solid‐enhancing compartments under CCNU, indicates that this cloudy‐enhancing compartment is tumor‐associated rather than a therapy‐induced phenomenon. B. Representative ∆qT1 maps of patient No 1 (female, age 53) at TP0 (baseline), TP1, and TP5. Large tumor burden was surrounded by a large cloudy‐enhancing compartment which decreased (89% volume reduction) at first follow‐up (TP1) 6 weeks after starting therapy. No tumor progression occurred during the 13.8 months’ follow‐up and the volume of the solid‐enhancing compartment even decreased to 43% at TP5 (Fig. 2). The marked decrease in the cloudy‐enhancing compartment just after starting therapy indicated a good therapy response which resulted in a long PFS. C. Representative ∆qT1 maps of patient No 9 (male, age 59) at baseline (TP0), TP1, and TP5 under therapy (antipode to Fig. 5B). Cloudy‐enhancing compartment increased about 200% at first follow‐up (TP1) after starting therapy. In this patient, PFS was quite shorter (5.4 months) and solid tumor volume increased up to 90% at TP5. This patient also had new contrast‐enhancing lesions in the brain stem and spinal canal (not shown).
