# Supplementary material for: Quantitative T1‐mapping detects cloudy‐enhancing tumor compartments predicting outcome of patients with glioblastoma
Source: Cancer Med. 2016 Nov 28;6(1):89–99. doi: 10.1002/cam4.966 (PMC5269700; doi:10.1002/cam4.966)

Figure Supplemental

A. Representative ∆qT1 maps at baseline (TP0) and seven follow-ups (TP 1-7) of patient No 3 (male, age 67). Patient had a residual solid-enhancing tumor volume in the right central region, which increased continuously during therapy. The cloudy-enhancing compartment firstly occurred at TP1 and increased again at TP2. During follow-up, the cloudy-enhancing compartment persisted and the solid-enhancing compartment increased steadily until therapy was intensified with CCNU at week 33 (marked with * after TP6). Its persistence during tumor progression, and its simultaneous decrease with the solid-enhancing compartments under CCNU, indicates that this cloudy-enhancing compartment is tumor-associated rather than a therapy-induced phenomenon.

B. Representative ∆qT1 maps of patient No 1 (female, age 53) at TP0 (baseline), TP1 und TP5. Large tumor burden was surrounded by a large cloudy-enhancing compartment which decreased (89% volume reduction) at first follow-up (TP1) 6 weeks after starting therapy. No tumor progression occurred during the 13.8 months’ follow-up and the volume of the solid-enhancing compartment even decreased to 43% at TP5 (Fig. 2). The marked decrease of the cloudy-enhancing compartment just after starting therapy indicated a good therapy response which resulted in a long PFS.

C. Representative ∆qT1 maps of patient No 9 (male, age 59) at baseline (TP0), TP1 and TP5 under therapy (antipode to Fig. 5B). Cloudy-enhancing compartment increased about 200% at first follow-up (TP1) after starting therapy. In this patient, PFS was quite shorter (5.4 months) and solid tumor volume increased up to 90% at TP5. This patient also had also new contrast enhancing lesions in the brain stem and spinal canal (not shown).


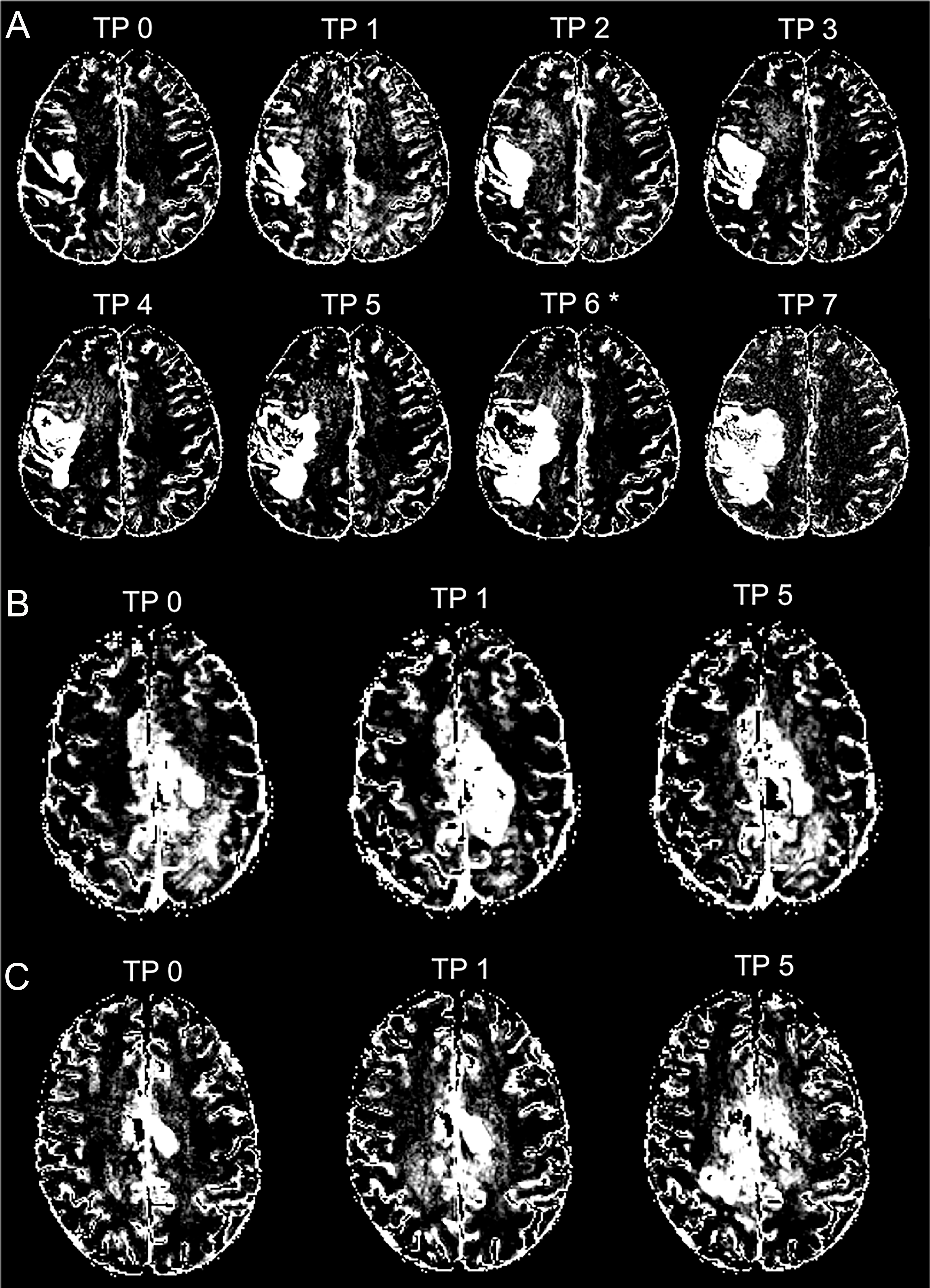

Supplement: Supplementary file 1 — Figure S1. A. Representative ∆qT1 maps at baseline (TP0) and seven follow‐ups (TP 1‐7) of patient No 3 (male, age 67). Patient had a residual solid‐enhancing tumor volume in the right central region, which increased continuously during therapy. The cloudy‐enhancing compartment firstly occurred at TP1 and increased again at TP2. During follow‐up, the cloudy‐enhancing compartment persisted and the solid‐enhancing compartment increased steadily until therapy was intensified with CCNU at week 33 (marked with * after TP6). Its persistence during tumor progression, and its simultaneous decrease with the solid‐enhancing compartments under CCNU, indicates that this cloudy‐enhancing compartment is tumor‐associated rather than a therapy‐induced phenomenon. B. Representative ∆qT1 maps of patient No 1 (female, age 53) at TP0 (baseline), TP1, and TP5. Large tumor burden was surrounded by a large cloudy‐enhancing compartment which decreased (89% volume reduction) at first follow‐up (TP1) 6 weeks after starting therapy. No tumor progression occurred during the 13.8 months’ follow‐up and the volume of the solid‐enhancing compartment even decreased to 43% at TP5 (Fig. 2). The marked decrease in the cloudy‐enhancing compartment just after starting therapy indicated a good therapy response which resulted in a long PFS. C. Representative ∆qT1 maps of patient No 9 (male, age 59) at baseline (TP0), TP1, and TP5 under therapy (antipode to Fig. 5B). Cloudy‐enhancing compartment increased about 200% at first follow‐up (TP1) after starting therapy. In this patient, PFS was quite shorter (5.4 months) and solid tumor volume increased up to 90% at TP5. This patient also had new contrast‐enhancing lesions in the brain stem and spinal canal (not shown). [file CAM4-6-89-s001.docx]
